# RETRACTION: Effects of Evidence‐Based Care on Diabetic Foot Ulcers: A Meta‐Analysis

**DOI:** 10.1111/iwj.70323

**Published:** 2025-03-06

## Abstract

**RETRACTION:** SunC.‐Z. , HeD.‐J. , CaoS.‐H. , QiY.‐H. , “Effects of Evidence‐Based Care on Diabetic Foot Ulcers: A Meta‐Analysis,” International Wound Journal 21, no. 1 (2024): e14403, 10.1111/iwj.14403.37735819 PMC10788586

The above article, published online on 21 September 2023, in Wiley Online Library (http://onlinelibrary.wiley.com/), has been retracted by agreement between the journal Editor in Chief, Professor Keith Harding; and John Wiley & Sons Ltd. Following an investigation by the publisher, all parties have concluded that this article was accepted solely on the basis of a compromised peer review process. The editors have therefore decided to retract the article. The authors did not respond to our notice regarding the retraction.

**RETRACTION:** C.‐Z. Sun , D.‐J. He , S.‐H. Cao , Y.‐H. Qi , “Effects of Evidence‐Based Care on Diabetic Foot Ulcers: A Meta‐Analysis,” International Wound Journal 21, no. 1 (2024): e14403, 10.1111/iwj.14403.37735819 PMC10788586

The above article, published online on 21 September 2023, in Wiley Online Library (http://onlinelibrary.wiley.com/), has been retracted by agreement between the journal Editor in Chief, Professor Keith Harding; and John Wiley & Sons Ltd. Following an investigation by the publisher, all parties have concluded that this article was accepted solely on the basis of a compromised peer review process. The editors have therefore decided to retract the article. The authors did not respond to our notice regarding the retraction.

